# Increased vulnerability of nigral dopamine neurons after expansion of their axonal arborization size through D2 dopamine receptor conditional knockout

**DOI:** 10.1371/journal.pgen.1008352

**Published:** 2019-08-26

**Authors:** Nicolas Giguère, Benoît Delignat-Lavaud, Freja Herborg, Aurore Voisin, Yuan Li, Vincent Jacquemet, Madhu Anand-Srivastava, Ulrik Gether, Bruno Giros, Louis-Éric Trudeau

**Affiliations:** 1 Departments of pharmacology and physiology, Department of neurosciences, Central Nervous System Research Group (GRSNC), Faculty of Medicine, Université de Montréal, Québec, Canada; 2 Molecular Neuropharmacology and Genetics Laboratory, Department of Neuroscience, Faculty of Health and Medical Sciences, University of Copenhagen, Copenhagen, Denmark; 3 Department of pharmacology and physiology, Faculty of Medicine, Université de Montréal, Québec, Canada; 4 Department of pharmacology and physiology, Research Center of the Hôpital de Sacré-Coeur de Montréal, Montréal, Québec, Canada; 5 Department of Psychiatry, McGill University Faculty of Medicine, Douglas Mental Health University Institute, Montreal, Québec, Canada; Florey Institute of Neuroscience and Mental Health, AUSTRALIA

## Abstract

Parkinson’s disease (PD) is a neurodegenerative disorder characterized by the loss of dopamine (DA) neurons in the substantia nigra pars compacta (SNc). Rare genetic mutations in genes such as Parkin, Pink1, DJ-1, α-synuclein, LRRK2 and GBA are found to be responsible for the disease in about 15% of the cases. A key unanswered question in PD pathophysiology is why would these mutations, impacting basic cellular processes such as mitochondrial function and neurotransmission, lead to selective degeneration of SNc DA neurons? We previously showed *in vitro* that SNc DA neurons have an extremely high rate of mitochondrial oxidative phosphorylation and ATP production, characteristics that appear to be the result of their highly complex axonal arborization. To test the hypothesis *in vivo* that axon arborization size is a key determinant of vulnerability, we selectively labeled SNc or VTA DA neurons using floxed YFP viral injections in DAT-cre mice and showed that SNc DA neurons have a much more arborized axon than those of the VTA. To further enhance this difference, which may represent a limiting factor in the basal vulnerability of these neurons, we selectively deleted in mice the DA D2 receptor (D2-cKO), a key negative regulator of the axonal arbour of DA neurons. In these mice, SNc DA neurons have a 2-fold larger axonal arborization, release less DA and are more vulnerable to a 6-OHDA lesion, but not to α-synuclein overexpression when compared to control SNc DA neurons. This work adds to the accumulating evidence that the axonal arborization size of SNc DA neurons plays a key role in their vulnerability in the context of PD.

## Author summary

Parkinson’s disease motor symptoms have been linked to age-dependent degeneration of a class of neurons in the brain that release the chemical messenger dopamine. The reason for the selective loss of these neurons represents a key unsolved mystery. One hypothesis is that the neurons most at risk in this disease are those with the most extensive and complex connectivity in the brain, which would make these cells most dependent on high rates of mitochondrial energy production and expose them to higher rates of oxidative stress. Here we selectively deleted in dopamine neurons a key gene providing negative feedback control of the axonal arbor size of these neurons, in the objective of producing mice in which dopamine neurons have more extensive connectivity. We found that deletion of the dopamine D2 receptor gene in dopamine neurons leads to dopamine neurons with a longer and more complex axonal domain. We also found that in these mice, dopamine neurons in a region of the brain called the substantia nigra show increased vulnerability to a neurotoxin often used to model Parkinson’s disease in rodents. Our findings provide support for the hypothesis that the scale of a neuron’s connectivity directly influences its vulnerability to cellular stressors that trigger Parkinson’s disease.

## Introduction

PD is a neurodegenerative disorder primarily characterized by a massive loss of DA neurons in the SNc that is also thought to be accompanied by the loss of other types of neurons in a select subset of brain regions including the locus coeruleus and the pedunculopontine nucleus [1]. Canonical symptoms include a range of motor deficits, but PD patients also often suffer from non-motor symptoms including olfactory deficits and constipation. Inherited mutations in gene products such as Parkin, Pink1, DJ-1, α-synuclein, LRRK2 or GBA are found in approximately 15% of cases. These gene products are involved in basic cellular processes including mitophagy, oxidative stress handling, mitochondrial antigen presentation, vesicular trafficking and lysosomal function. One of the key unanswered questions in PD research is why alterations in such ubiquitous processes lead to selective degeneration of a select subset of neuronal populations in the brain including SNc DA neurons. A striking example of this selectivity is the much higher resilience of the neighboring DA neurons of the ventral tegmental area (VTA), which are far less affected than SNc DA neurons in PD [1]. In the last few decades, many hypotheses have been raised about the core characteristics of SNc neurons that are responsible for their large bioenergetic requirements and that could explain their selective vulnerability. These include, but are not limited to, pacemaking activity [2], high DA- and iron-related toxicity [3,4] and possessing a highly elaborate, long-range axonal arborization [5–8]. All these characteristics are thought to exert an important pressure on the capacity of these cells to efficiently produce energy and cope with the associated oxidative stress. In this context, any other subsequent cellular stresses associated with some of the genetic alterations mentioned above, as well as aging and exposure to environmental toxins could trigger the disease. We have previously showed *in vitro* that SNc DA neurons have a higher basal rate of mitochondrial oxidative phosphorylation and ATP production and a smaller reserve capacity compared with the less vulnerable DA neurons of the VTA, characteristics that appear to be the result of the highly complex axonal arborization of these neurons [8]. We therefore postulated that the size of this axonal arborization might be a significant contributor to the differential vulnerability of SNc and VTA DA neurons in PD.

Based on our previous work and on modelling of the impact of the axonal arborization size on energy requirements [9,8], it is possible that the relatively small size of the axonal arborization of mouse DA neurons compared to humans (10 fold smaller) could explain the apparently high resilience of mouse DA neurons and the associated difficulty to produce optimal animal models in this species. Indeed, mouse models with genetic deletions of the key genes found in familial forms of the disease generally do not present age-dependent neuronal loss [10–14]. If the smaller axonal arborization size of mouse DA neurons is a key limiting factor for their vulnerability, it might be possible to increase this vulnerability by increasing their axonal arborization size *in vivo*. In order to reach this objective and test our hypothesis, we generated mice with a conditional deletion of the DA D2 receptor in DA neurons (D2-cKO). Increased DA terminal density has been suggested to occur under chronic D2 antagonist administration [15,16] and in the constitutive knockout model of this receptor [17] and D2 agonists have been shown to reduce the density of axon terminals established by DA neurons [15,18]. Here we surmised that a cell-specific knockout of this receptor in DA neurons should lead to an increased size and complexity of the axonal arborization of these neurons and increase their intrinsic vulnerability.

We find that in the intact mouse brain, the axonal arborization size of SNc DA neurons is 3-fold larger than that of less vulnerable VTA DA neurons. We further demonstrate that in D2-cKO mice, the axonal arborization size of SNc DA neurons is 2-fold larger relative to control mice, a phenotype associated with impaired evoked DA release and increased vulnerability to 6-OHDA, but not to α-synuclein overexpression. This work provides strong evidence in favor of the hypothesis that the axonal arborization size of SNc DA neurons plays a key role in regulating their basal vulnerability in the context of PD.

## Results

### SNc DA neurons have a much more elaborated striatal axonal arborization than VTA DA neurons in the mouse brain

**Fig 1:**
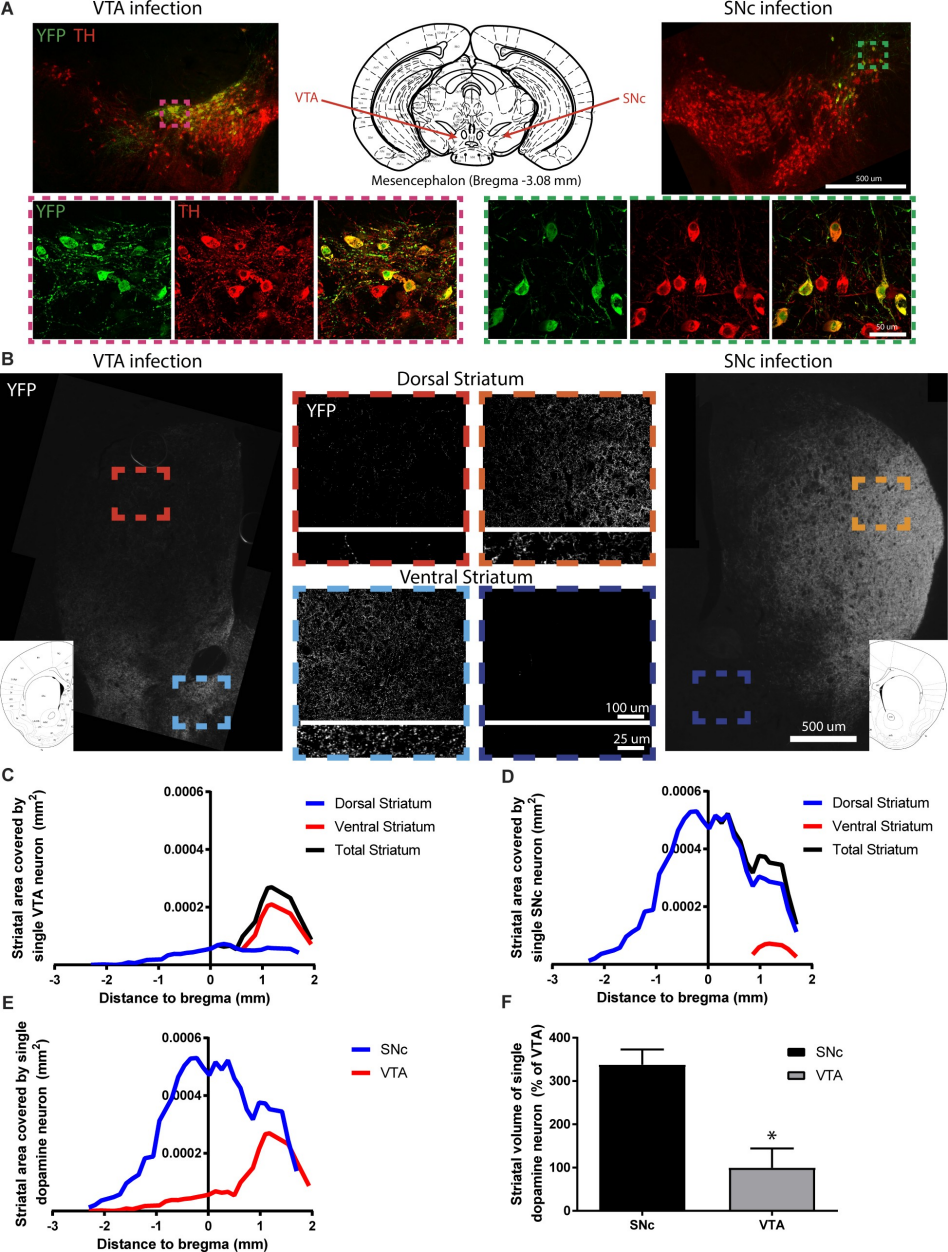
SNc DA neurons have a longer axonal arborization than VTA DA neurons *in vivo*. Axonal arborization was estimated by performing AAV injections in adult DAT-Cre mice to conditionally express eYFP in either VTA or SNc DA neurons and visualized by immunofluorescent labelling for eYFP and TH (A). The extent of the axonal arborization was then observed in the striatum (B) and measured thought-out the ventral and dorsal striatum for VTA (C) and SNc (D) targeted injections. Direct (E) and relative (F) comparisons of the total striatal axonal arborization size was performed. N = 3 brains/group, mean ± SEM, *p>0.05. Brain schematics modified from The Mouse Brain in Stereotaxic Coordinates 3rd Edition by George Paxinos and Keith B.J. Franklin [73].

If axonal arborization size is a critical determinant of the selective vulnerability of SNc DA neurons, a prediction is that the axonal domain of these neurons should be more arborized than that of the more resilient VTA DA neurons *in vivo*. Since there is no specific axonal marker to distinguish between SNc and VTA DA neurons, we injected a small amount of floxed AAV2-eYFP in the SNc or the VTA of adult DAT^Cre/+^ mice to label a few hundred (~300–1000) DA neurons from one or the other population (Fig 1A). For more examples of SNc targeted infections, see. S1 Fig. After immunolabeling and manual counting of the infected neurons, we quantified the extent of the related axonal arborization within the striatum using confocal imaging to systematically sample images from slices throughout the rostro caudal axis (Fig 1B). As expected, the majority of SNc DA neurons projections were found in the dorsal striatum and the majority of VTA DA neurons projections were found in the ventral striatum. We next extrapolated the arborization density obtained from each slice to the size of the striatum on that slice and normalized it by the number of infected neurons. Finally, we plotted the arborization area obtained as a function of bregma coordinates for VTA (Fig 1C) and SNc (Fig 1D) targeted infections. Comparing the extent of the total arborization revealed a 3-fold larger axonal arbour for SNc compared to VTA DA neurons (Fig 1E and 1F).

### Increase in DAT but not TH striatal expression in D2-cKO without change in the number of neurons

**Fig 2:**
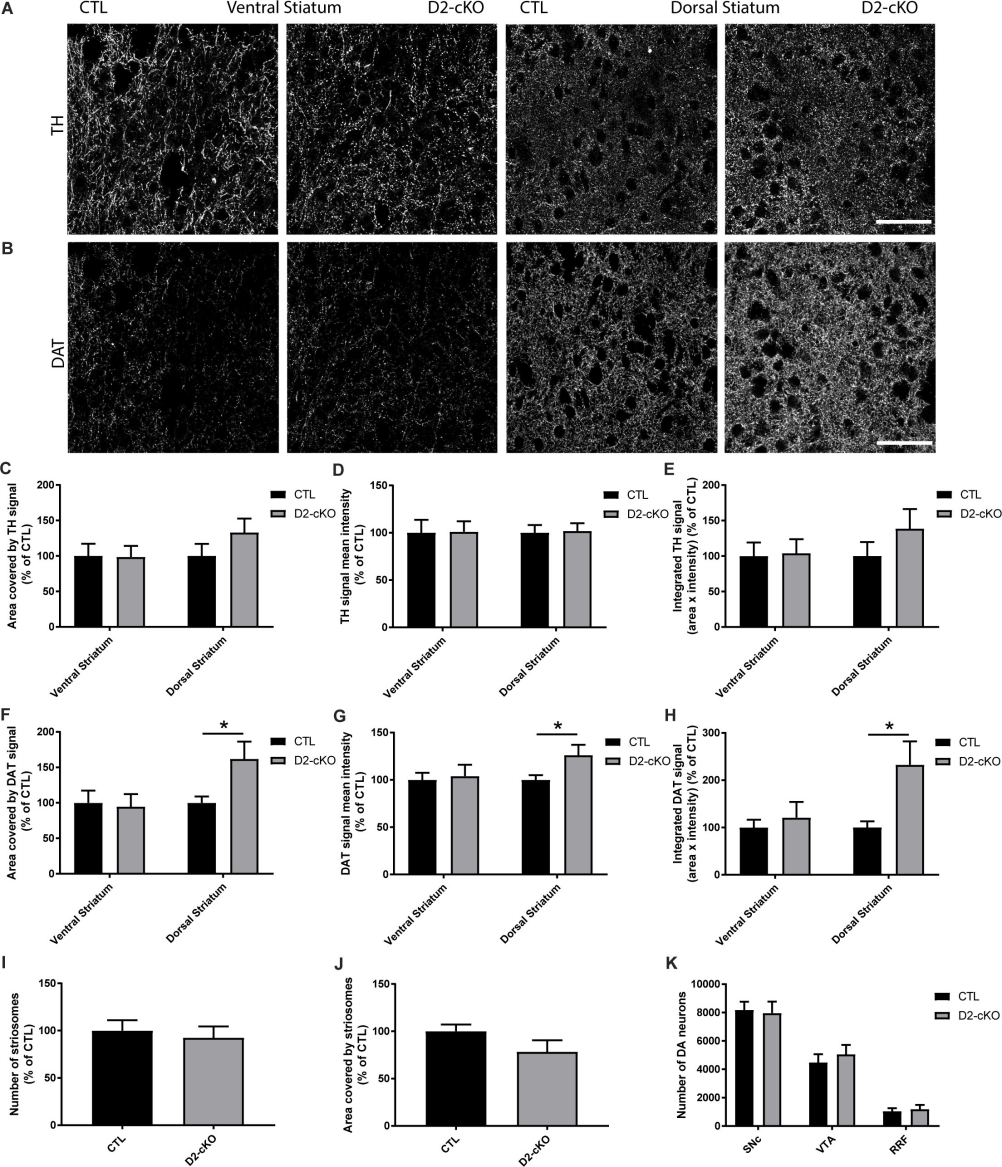
Increase in DAT but not TH striatal expression in D2-cKO mice, without a change in the number of DA neurons. TH (A) and DAT (B) immunofluorescence levels were measured in the ventral and dorsal striatum in controls and D2-cKO mice. Scale bar = 50 μm. % area covered (C,F), mean signal intensity (D,G) and integrated signal (E,H) were quantified for TH and DAT signals as well as the number of striosomes (I) and their size (J) using DAT signal. N = 15–22 brains/group, mean ± SEM, *p>0.05. The number of DA neurons in the SNc, VTA and RRF (K) was measured using stereological counting. N = 4–5 brains/group, mean ± SEM.

Because an increase in axonal arbour size could increase the vulnerability of DA neurons, we aimed at increasing the axonal arborization of DA neurons by selective genetic deletion of the DA D2 receptor. To do so, we crossed DAT^IRES-Cre^ mice with Drd2^loxP^ mice and generated DAT^IRES-Cre/+^; Drd2^loxP/loxP^ mice as previously described [19]. Control mice were heterozygotes for Cre expression (DAT^IRES-Cre/+^; Drd2^+/+^). In these D2-cKO adult mice, we examined the axonal varicosities of DA neurons by measuring TH and DAT immunolabeled structures using confocal imaging in the ventral and dorsal striatum (Fig 2A and 2B). We observed no change in the area covered by TH signal (Fig 2C), the TH mean signal intensity (Fig 2D) or total TH signal (Fig 2E) in any part of the striatum. However, we observed an increased area covered by the DAT signal in the dorsal striatum (Fig 2F) with an increased DAT signal intensity (Fig 2G), which resulted in a more than 2-fold increase in total DAT signal (Fig 2H). No changes were observed in the ventral striatum. This increased DAT signal in the dorsal striatum was not the result of changes in the number (Fig 2I) or size (Fig 2J) of striosomes and was not a result of an increased number of DA neurons in the SNc, VTA or retrorubral field (RRF), as determined by unbiased stereological counting (Fig 2K).

### Increased axonal arborization size of SNc but not VTA DA neurons in D2-cKO mice

**Fig 3:**
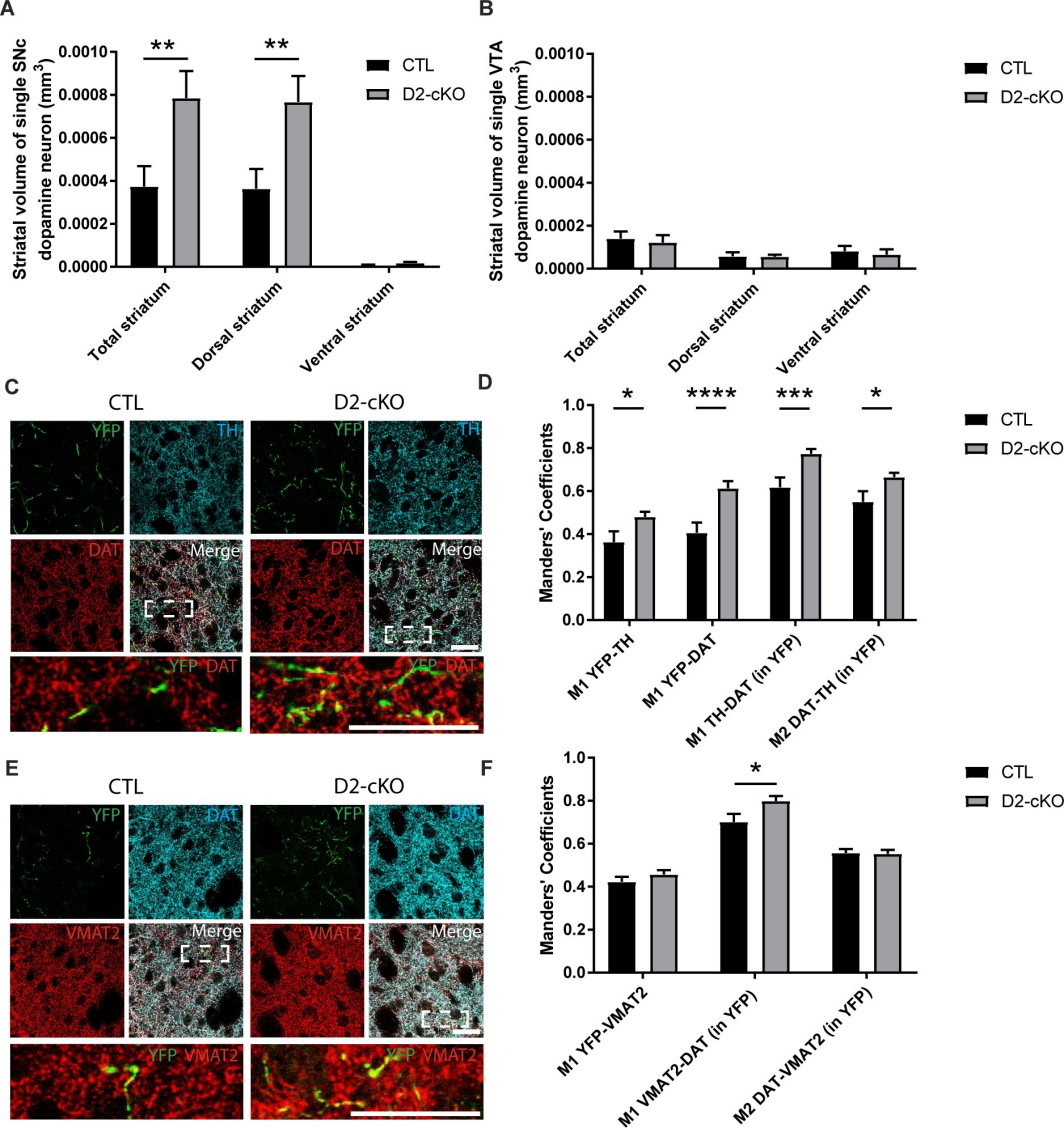
Increased axonal arborization size of SNc but not VTA DA neurons in D2-cKO mice. The axonal arborization of SNc or VTA DA neurons was selectively visualized using AAV injections in adult DAT^IRES-Cre/+^;DRD2^LOX/LOX^ mice to express eYFP in either SNc or VTA DA neurons. The extent of the axonal arborization was observed in the striatum and measured thoughout the ventral and dorsal striatum for SNc (A) and VTA (B) targeted injections. N = 6–10 brains/group, mean ± SEM, **p>0.01. Colocalization of YFP-positive SNc dopaminergic axonal processes (C) with TH and DAT, and the colocalization of TH and DAT were measured with Mander’s coefficients (M1 and M2) (D). Colocalization of YFP-positive SNc dopaminergic axonal processes (E) with VMAT2 and colocalization of VMAT2 and DAT were measured with Mander’s coefficients (F). M1 = proportion of signal 1 that colocalize with signal 2, M2 = proportion of signal 2 that colocalize with signal 1. Scale bar = 80 μm. N = 24–35 images/group, mean ± SEM, *p>0.05, ***p>0.001, ****p>0.0001.

To confirm that this increased dorsal striatal DAT signal was the result of an increase in the axonal arborization size of SNc DA neurons, we again used conditional viral labelling to visualize the axonal domain of SNc and VTA DA neurons in D2-cKO mice. We observed a 2-fold increase in the axonal arborization size of SNc DA neurons in D2-cKO mice (Fig 3A), with no change for VTA DA neurons (Fig 3B). Comparing axonal arborization size of SNc and VTA DA neurons from control mice again showed a 3-fold difference between the two populations (Fig 3A vs 3B). To better characterize this expanded axonal arbour originating from SNc D2-cKO DA neurons, we next measured the level of colocalization of virally-expressed YFP with DAT or TH (Fig 3C). There was an increased colocalization of TH or DAT with the YFP-labelled axonal varicosities of D2-cKO mice and a general increased colocalization of TH and DAT inside these processes (Fig 3D). To evaluate if these new processes were likely to be functional and able to release DA, we measured the colocalization of VMAT2 with YFP (Fig 3E) and found it to be unchanged (Fig 3F). We also found an increased colocalization of VMAT2 and DAT inside these processes.

### Reduced striatal DA release in D2-cKO mice without changes in DA reuptake kinetics or in striatal surface DAT levels

**Fig 4:**
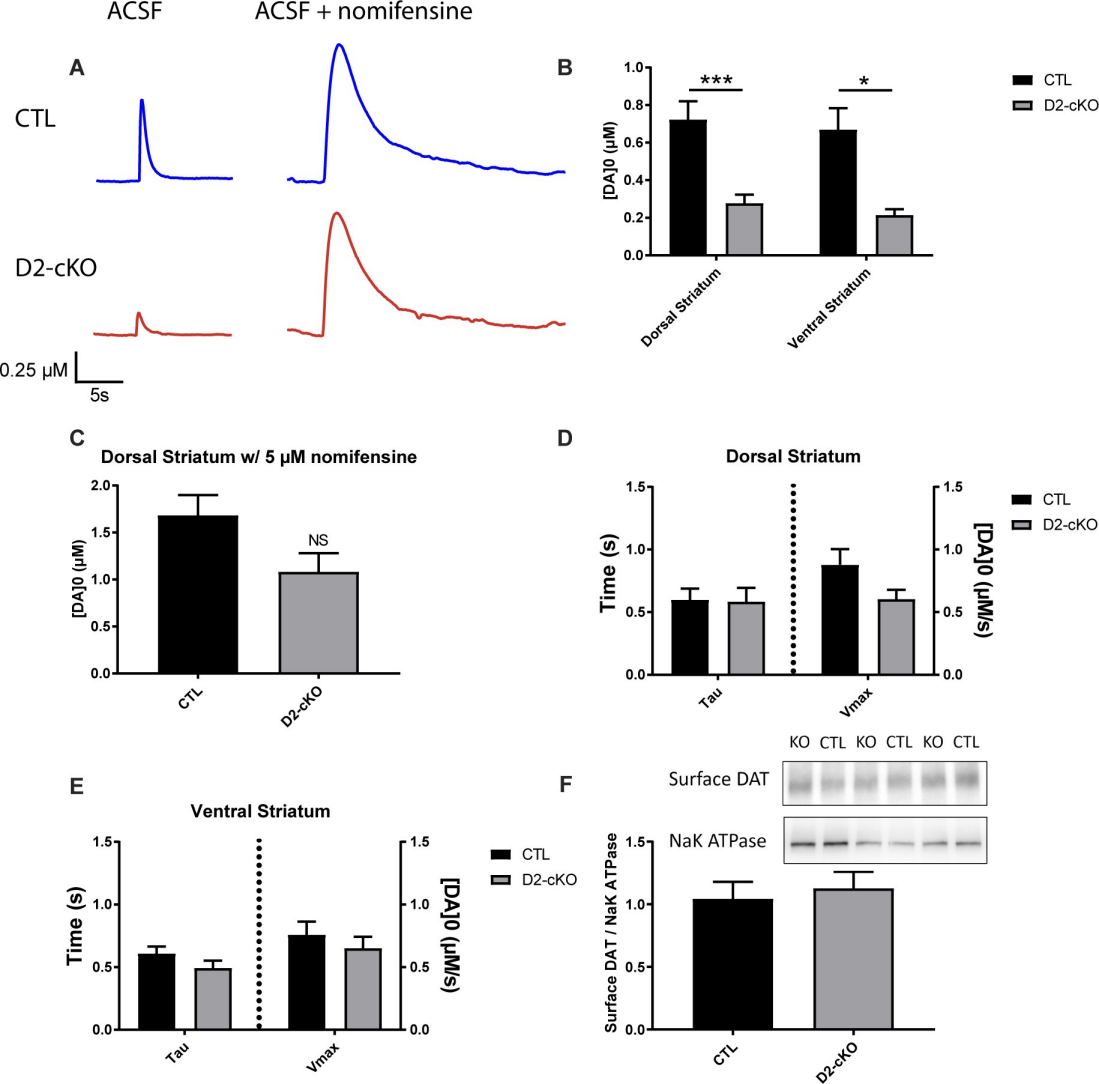
Decrease in striatal DA release in D2-cKO mice without changes in DA reuptake kinetics or in striatal surface DAT levels. Cyclic voltammetry was used to measure the amount of DA released during single electrical stimuli on acute brain slices (A) in the ventral and dorsal striatum (B). Single stimulations were also used in the presence of the DAT antagonist nomifensine (C). DA reuptake kinetics were extracted from recordings obtained in response to single pulse stimulation by fitting an exponential curve based on the Michaelis-Menten equation for the dorsal (D) and ventral (E) striatum. N = 8–9 brains/group, mean ± SEM, *p>0.05, ***p>0.001. Surface biotinylation assay was performed on the striatum of a separate cohort of control and D2-cKO mice (F). Biotinylated surface DAT band intensities were normalized to Na^+^/K^+^-ATPase band intensities using ImageJ gel analysis software. N = 10 brains/group, mean ± SEM.

An increased density of dopaminergic axonal fibers in the striatum, as well as the genetic removal of the pre-synaptic D2R known to control DA synthesis and release, could lead to increased DA release. Alternately, the enhanced bioenergetic requirements associated with a broader axonal arbour could lead to impaired DA neurotransmission. To distinguish between these possibilities, we quantified DA release evoked by single electrical pulses in acutely prepared striatal brain sections from D2-cKO and control mice using fast-scan cyclic voltammetry (Fig 4A). We found that DA release was significantly reduced in the dorsal and ventral striatum (Fig 4B). However, this difference was greatly diminished following incubation with the DAT antagonist nomifensine (Fig 4C). This observation of a partial rescue with nomifensine, coupled with our observation of increased striatal DAT immunoreactivity (Fig 2C–2E) could imply that increased DAT function in D2-cKO mice was the cause of the reduced activity-dependent DA overflow. Alternately, as DAT blockers including nomifensine and cocaine have been reported to also promote DA release though other mechanisms [20–22], the apparent rescue could result from an enhancement of DA release and not reuptake. To distinguish between these two possibilities, we examined the kinetics of DA release. Comparing D2-cKO and control mice, we found no change in kinetics of DA reuptake (tau) or in the maximal rate of reuptake (Vmax) in the dorsal (Fig 4D), or ventral (Fig 4E) striatum, suggesting no robust change in DAT function in D2-cKO mice. To further address this issue, we also performed a surface biotinylation assay from the striatum of a separate cohort of control and D2-cKO mice and confirmed that there were no significant changes in surface DAT levels in the striatum in the absence of D2 autoreceptors (Fig 4F).

### SNc DA neurons from D2-cKO are more vulnerable to 6-OHDA but not to α-synuclein overexpression

**Fig 5:**
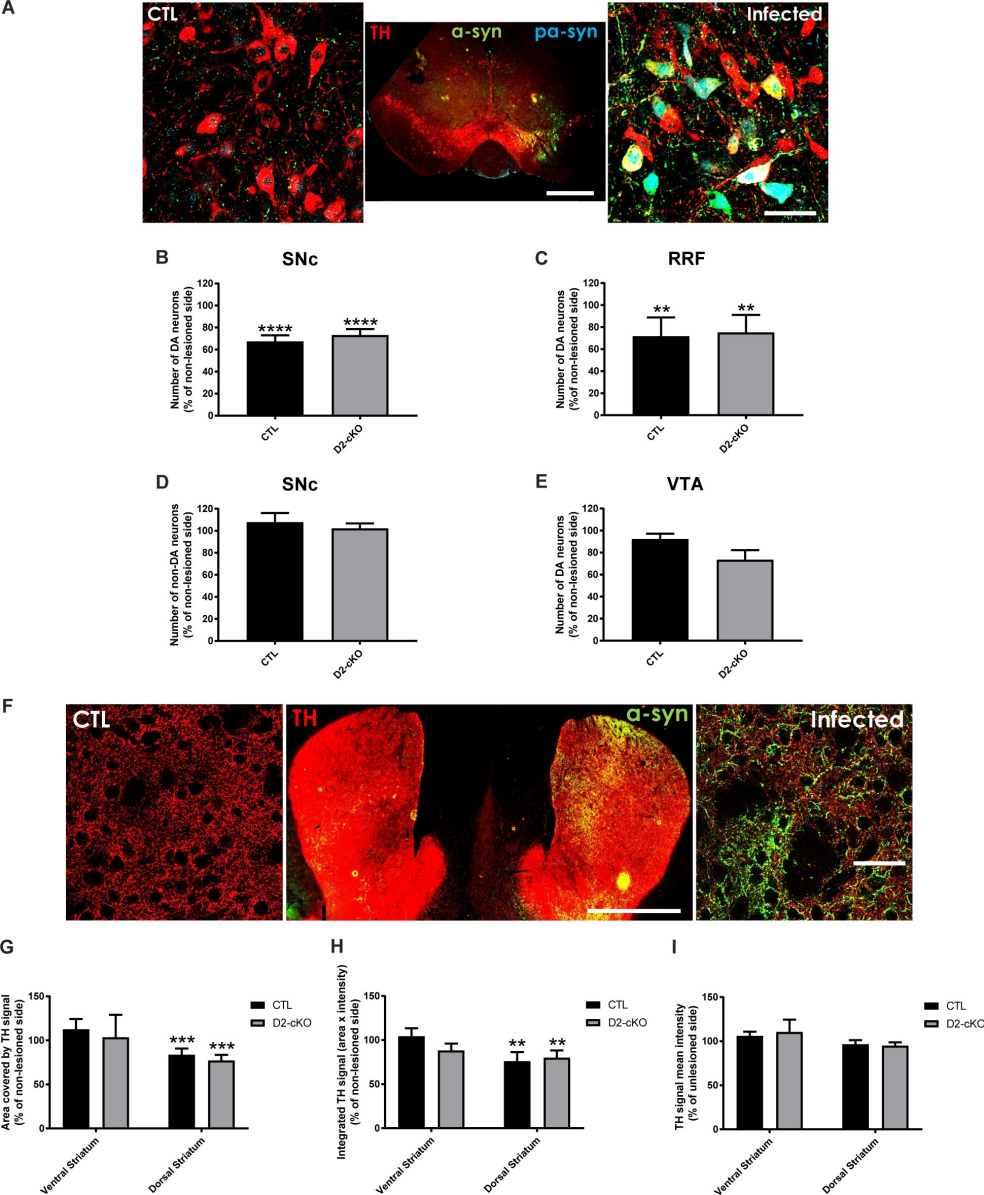
SNc DA neurons from D2-cKO are not more vulnerable to α-synuclein overexpression. α-synuclein viral overexpression in the mesencephalon was used to evaluate the impact of the increased axonal arborization size of D2-cKO SNc DA neurons on their vulnerability. In the mesencephalon (A), SNc TH+ (B), RRF TH+ (C), SNc TH- (D) and VTA TH+ neurons (E) were counted using stereological methods. N = 9 brains/group, mean ± SEM, ** p>0.01, **** p>0.0001. Scale bars = 1mm and 50 μm. In the dorsal and ventral striatum (F), TH signal area (G), mean signal intensity (H) and integrated signal (I) were measured. N = 9–12 brains/group, mean ± SEM, ** p>0.01, *** p>0.001. Scale bars = 1mm and 50 μm.

As an increase in axonal arbour size in D2-cKO SNc DA neurons is predicted to induce a larger bioenergetic burden on these neurons, we next examined their vulnerability in two different mouse models of PD: the α-synuclein viral overexpression model and the intra-striatal 6-OHDA model. AAV-mediated wild-type α-synuclein overexpression was achieved by stereotaxic injection into the mesencephalon (Fig 5A). Three months after virus injection, stereological counting revealed a loss of 25–35% of DA neurons in the SNc and RRF (Fig 5B and 5C), with no change in the number of non-DA neurons (Fig 5D) and no significant change in the VTA (Fig 5E). This cell loss in the SNc and RRF was not significantly different in D2-cKO mice compared to control mice. We also observed the presence of phosphorylated α-synuclein positive cell bodies (Fig 5A), a good indicator of the toxicity induced by the overexpression. In the dorsal striatum only (Fig 5F), we observed a small 20% decrease in TH signal area (Fig 5G) and total signal (Fig 5H) with no change in signal intensity (Fig 5I).

At the behavioral level, mice overexpressing α-synuclein only showed a modest increased preference for ipsilateral paw use (S2A Fig), with no change in the total number of steps and no difference between D2-cKO and CTL mice (S2B Fig). In the rotation test, neither basal nor amphetamine-induced rotational preferences were altered (S2C and S2D Fig), with amphetamine inducing an expected increase in total number of rotations (S2E Fig). These finding are in keeping with the modest level of cell loss and striatal denervation in this model.

**Fig 6:**
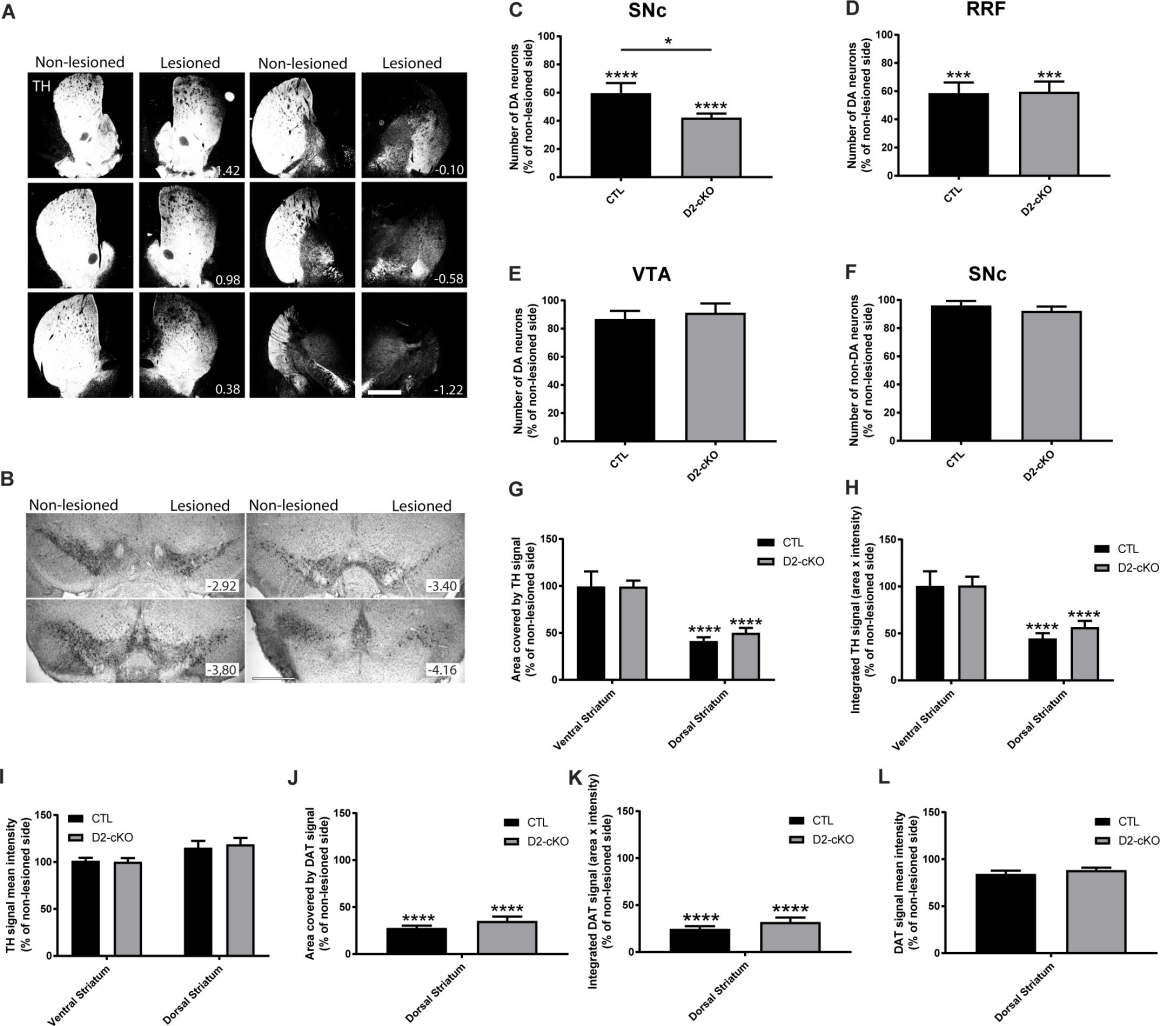
SNc DA neurons from D2-cKO mice are more vulnerable to 6-OHDA. The 6-OHDA partial lesion model was used to evaluate the impact of the increased axonal arborization size of D2-cKO SNc DA neurons on their vulnerability. (A) TH immunohistochemistry illustrating the density of dopaminergic innervation at different coordinates along the rostro-caudal axis of the striatum in a CTL mouse. Scale bar = 1 mm. (B) TH immunohistochemistry was used to localize and count the number of DA neurons in the SNc, RRF and VTA. Quantification of the survival of TH+ SNc (C), RRF (D), VTA (E) and TH- SNc neurons (F) was performed using stereological counting methods. N = 10–11 brains/group, mean ± SEM, *p>0.05, *** p>0.001, **** p>0.0001. The signal area (J), total area (H) and signal intensity (I) of TH (G)was measured in the dorsal and ventral striatum. DAT was also quantified in the dorsal striatum (J, K, L). N = 6–8 brains/group, mean ± SEM, **** p>0.0001.

We next examined the vulnerability of DA neurons using a second, different model of PD using the DA neuron-specific toxin 6-OHDA. Unilateral injection in the dorsal striatum at a low dose (1.5μg in 0.5 μL) (Fig 6A) was performed in order to produce a partial loss of dopaminergic cell bodies (Fig 6B). In control mice, one month after the 6-OHDA lesion, we measured an approximate 40% loss of DA neurons in the SNc (Fig 6C) and the RRF (Fig 6D), with no significant loss in the VTA (Fig 6E) or for non-DA of the SNc neurons (Fig 6F). Interestingly, in the D2-cKO mice, approximately 60% of SNc DA neurons were lost, representing almost 50% more neurodegeneration than for control mice (60% loss vs 42% loss for CTL) (Fig 6C). As for axon terminals, TH signal area (Fig 6G) and total TH signal (Fig 6H) were both reduced by approximately 50% in the dorsal striatum, with no change detected in the ventral striatum, confirming the specificity of the lesion. In addition, DAT signal area (Fig 6J) and total signal (Fig 6K) were reduced by approximately 75% in the dorsal striatum. There were no significant changes in TH and DAT signal intensity (Fig 6I and 6L), suggesting loss of axonal terminals rather than simply reduced TH and DAT levels. Even if more neurons were lost in the SNc in D2-cKO mice compared to control mice, no significant difference was observed between the two genotypes (Fig 6G–6L) at the terminal level, compatible with compensatory axonal sprouting.

**Fig 7:**
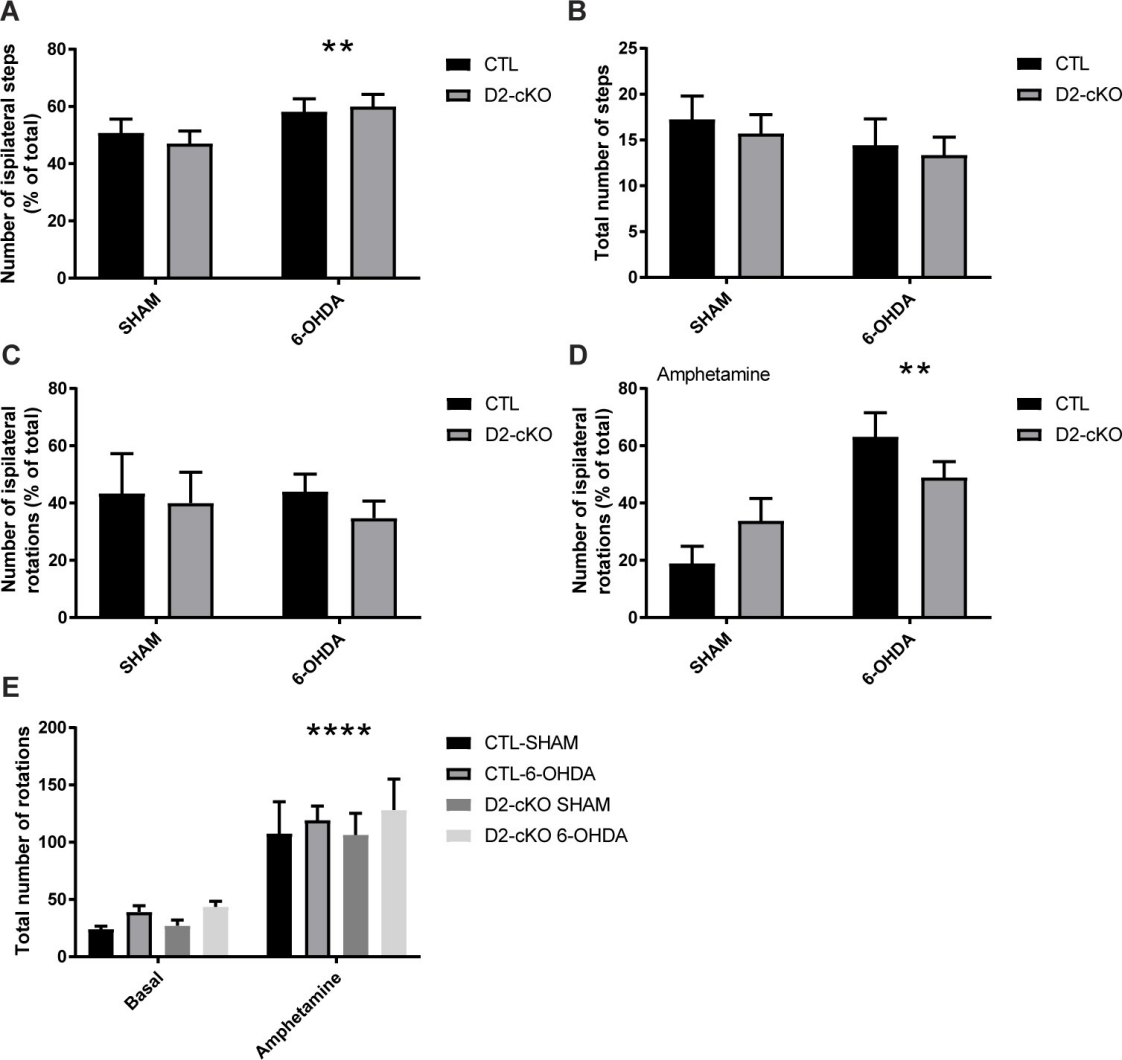
Behavioral changes following 6-OHDA injection. Paw preference (A) and total number of steps (B) were measured during a stepping test. Rotational behaviour was assessed at basal state (C) and after amphetamine administration (D). Total number of rotations were also measured (E). N = 4–11 brains/group, mean ± SEM, ** p>0.01, **** p>0.0001.

In line with the modest decrease in TH signal within the striatum of these mice and the absence of genotype effect in striatal denervation, we failed to detect a difference between D2-cKO mice and controls in motor behaviors (Fig 7). However, the 6-OHDA lesion caused an increased preference for the ipsilateral paw in the stepping test (Fig 7A) with no change in total number of steps (Fig 7B). In the rotation test, we observed no changes in rotational preference at basal levels (Fig 7C), but an increased preference for ipsilateral rotations under amphetamine was detected (Fig 7D). Finally, the total number of rotations was significantly increased following amphetamine administration (Fig 7E).

## Discussion

One of the key unanswered questions in PD research is why DA neurons of the SNc are particularly vulnerable. In the last few decades, a number of hypotheses have been raised regarding the core characteristic responsible for this vulnerability, including DA and iron toxicity, pacemaking activity and the establishment of a large and complex axonal arborization [7,3,4,23]. One commonality between these features is that they all lead to increased oxidative stress and bioenergetic demands, that are easily destabilized in pathology. Compatible with this model, we previously showed *in vitro* that SNc DA neurons have a higher rate of mitochondrial oxidative phosphorylation and basal oxidative stress compared with less vulnerable DA neurons of the VTA, characteristics that appear to be the result of their highly complex axonal arborization [8]. These results suggest that the size of this axonal arborization might be a critical determinant differentiating between surviving and degenerating neurons in PD.

### Relative axonal arborization size of VTA and SNc DA neurons

The size of the axonal arbour of SNc DA neurons was measured previously in the intact rat brain [24,25], but no direct comparison of this parameter with less vulnerable VTA DA neurons was available prior to the present work. However, by dividing the estimated number of terminals in the rat ventral and dorsal striatum with the corresponding number of DA neurons in the VTA and SNc, it had been previously estimated that SNc DA neurons have an 8-fold broader striatal axonal arborization compared to VTA DA neurons [7]. In the present work, we directly measured axonal arborization size of both neuronal populations in the entire striatum and similarly found a much larger axonal (3-fold) arborization for SNc compared to VTA DA neurons in mice. The smaller difference between our finding (3-fold) and the previous estimate (8-fold) could be due to the use of different species (rats vs mice), but we additionally took into account that VTA DA neurons also project to the dorsal striatum; projections which were not considered in the previous estimation [7]. The projections of VTA neurons to the dorsal striatum were much more diffuse, but because of the much larger size of the dorsal striatum compared to the ventral striatum, they accounted for a significant amount of the total number of projections from the VTA. These projections were also previously examined in a single neuron tracing study in mice [26], but in this work, the authors did not compare VTA to SNc neurons. They nonetheless confirmed that part of VTA DA neurons projections were outside of the ventral striatum, compatible with previous classical work describing mesocortical and mesolimbic pathways [27]. It is also possible that we underestimated the axonal arborization size of SNc DA neurons, since we were not able to selectively label neurons from the most ventral part of the SNc, who are known to be particularly vulnerable in PD [28], since they were too close to the VTA. It is possible that these highly vulnerable neurons could have an even broader axonal arborization.

Another potential caveat of this study is that we did not quantify axonal processes outside of the striatum, which could have led to an overestimated difference between SNc DA neurons and VTA DA neurons, since VTA neurons are known to also project to other brain regions such as the cortex, amygdala and septum. However, in initial experiments, a global evaluation of these regions revealed only a very low relative density of dopaminergic processes compared to the striatum. We thus limited our quantification to the striatum in the present study, which is the main projection site for both populations of DA neurons. Although this represents a limitation, we consider it unlikely that our estimates were significantly affected by this focus on striatal projections. In keeping with this possibility, the relative difference between the size of the axonal arborization of VTA and SNc DA neurons found in the present study is quite similar (2–3 fold larger for SNc compared to VTA) to what we previously observed *in vitro* [8]. Another limitation of the present work is that due to the quantity and volume of injected virus, we were not able to separately quantify the axonal arborization size of different subtypes of SNc or VTA DA neurons. Considering that the ventral tier of the SNc is much more vulnerable in PD compared to the dorsal tier [28] and that projections from the different subpopulations of DA neurons reach different subregions of the striatum [29], it would be of major interest in future work to examine axonal arborization size of different subpopulations of the SNc in relation to their differential vulnerability in PD. The use of intersectional genetic tools might be better suited than trying to reduce viral injection volume to tackle this question.

### Using D2-cKO to increase SNc DA neurons axonal arbour size

In the present study, we used D2-cKO mice to examine the vulnerability of DA neurons under conditions where these neurons develop an even larger axonal arborization. Increased DA terminal density in the dorsal striatum had been previously described in a constitutive [17] knockout model of this receptor. In order to focus on cell-autonomous mechanisms of vulnerability, we deleted the D2R gene selectively in DA neurons by crossing Drd2^loxP^ mice with DAT^IREScre^ mice. Using these DAT^IREScre^/Drd2^loxP^mice, we surprisingly did not find any changes in TH signal in the striatum, an observation that could reflect the highly plastic and homeostatic nature of TH expression in response to perturbations such as neurotoxins, which might make it somewhat unreliable to assess the extent of loss of axonal processes [30–36]. On the other hand, we did observe a significant increase in DAT immunoreactive processes in the dorsal striatum, as shown previously in the constitutive KO model [17], with no increase in the number of DA neurons. This is also similar to what has been observed previously in the hippocampus of this cKO model [19]. However, DAT expression and localisation can be altered by many mechanisms [37,38]. For example, this transporter is known to form protein-protein interactions with the D2 DA receptor, which is thought to promote DAT localisation to the plasma membrane [39–42]. For this reason, the lack of D2 receptors in our D2-cKO models could have altered the expression of DAT by compensatory mechanisms and not directly as a result of an increase in axonal arborization size.

To evaluate if the increase in DAT immunoreactive processes reflected an increase in axonal arborization size and was originating from SNc DA neurons and not from DA neurons from other regions such as the VTA, we took advantage of a viral labelling strategy to conditionally express a fluorescent reporter protein in SNc or VTA DA neurons. Doing so, we confirmed that SNc but not VTA DA neurons have an increased number of axonal processes in the striatum of the D2-cKO mice. To validate whether expanded axonal domains contained terminals that were likely to release DA, we also quantified the presence of TH, DAT and VMAT2 in these virally-labelled axonal processes. We found that there was an increase in colocalization with TH and DAT and no change in VMAT2 density in axonal processes, arguing that the increase in axonal size did not come at the expense of a loss in neurochemical identity.

We next used fast scan cyclic voltammetry to measure DA release in the striatum and to gain further insight into the functionality of dopaminergic axons in this model. We found a general decrease in DA release that was partially rescued in the presence of a DAT antagonist. Our finding of a decrease in evoked DA overflow, although somewhat counter-intuitive when considering the autoreceptor function of the D2 receptor, is in line with previous observations of constitutive or conditional D2R KO mice [43–47] (but see [48]). While we found here that this reduced DA release could be rescued by nomifensine, in a previous study, the use of a DAT antagonist was not sufficient to return DA levels to normal in the engrailed1-based D2-cKO [44]. It should be noted however that in this later work, while the control condition had both alleles of englailed1, the D2-cKO mice had only one allele of this transcription factor, which is otherwise critical for the development of DA neurons. Since knockout of even only one allele of engrailed1 has been shown to affect the number of DA neurons and the density of their terminals [49,50], it is possible that DA release in this model was affected by both the KO of the D2 receptor and the reduced engrailed1 expression as well as by the possible removal of the D2 receptor in engrailed expressing non-DA neurons of the VTA and SNc.

It is also important to note again that there have been reports that activation of D2 receptors in dopaminergic terminals regulates positively the localization of the DAT to the plasma membrane [39–42]. In our D2-cKO mice, although we detected an increase in DAT levels by immunofluorescence, we did not observe any significant change in reuptake kinetics as assessed from cyclic voltammetry recordings. We also did not detect a significant change in DAT surface levels using a DAT surface biotinylation assay. However, a reduction in Vmax has been observed in a previous study in which the D2 receptor was knocked down acutely using siRNA [47], although reuptake kinetics (tau) were not reported. The difference with our data could be explained by the acute nature of the deletion in this previous study. In the context of the absence of a change in reuptake kinetics, our finding of an apparent rescue of DA release in the presence of the DAT blocker nomifensine is puzzling. One possibility is that nomifensine was able to rescue a deficit in axon terminal function at a step which is independent from DAT activity. Previous work has indeed shown that DAT blockers including cocaine and nomifensine are able to enhance the exocytotic release of DA through a mechanism that is not yet clearly defined but that has been suggested to involve synapsin [20–22].

### Axonal arborization size as vulnerability factor

The goal of this work was to provide a first *in vivo* test of the importance of axonal arborization size on the vulnerability of SNc DA neurons. We confirm here that D2-cKO mice represent a model in which an expansion of the axonal arborization of SNc DA neurons can be detected. Based on our previous work performed with primary DA neurons [8], we predicted that this should lead to increased vulnerability of SNc DA neurons. In keeping with this hypothesis, we found that D2-cKO SNc DA neurons were more vulnerable to a 6-OHDA lesion initiated at the axon terminal level. An alternate interpretation of this increased neuronal loss in D2-cKO mice could be that the basal increase in DAT-positive varicosities observed in these mice led to an increased uptake of 6-OHDA. Although this possibility cannot be formally excluded, its likelihood is limited because our cyclic voltammetry reuptake kinetic measurements argue for an absence of change in DAT functionality at the plasma membrane, a finding that is in line with our observation of a lack of change in DAT surface levels in the striatum of D2 cKO mice. In the 6-OHDA model, we also observed a stronger loss of cell bodies than striatal terminals, with similar levels of striatal TH and DAT fiber density in D2-cKO mice compared to control mice. This finding argues for robust axonal sprouting from surviving neurons in the D2-cKO mice. This is in line with work showing presence of compensatory reinnervation in this lesion model [51,52] and is also supported by the absence of exacerbated 6-OHDA induced behavioral impairements in the D2-cKO mice. In future work, it would be of interest to look at the vulnerability of VTA DA neurons to 6-OHDA in the D2-cKO model using toxin injection targeted to the ventral striatum, as these neurons do not show any changes in their axonal arborization size, but are thought to participate in intense axonal spouting in this lesion model [51,53].

Because the D2 receptor regulates many cellular processes, we cannot completely exclude the possibility that lack of D2 receptors could have increased the vulnerability of SNc DA neurons through mechanisms other than the increased axonal arborization size. Future work will be required to determine the origins of this enhanced neuronal loss, but an increased level of basal oxidative stress in SNc DA neurons could be implicated and synergistically lead to sufficient oxidant stress to initiate apoptotic death of DA neurons [54–56]. ROS production induced by 6-OHDA has also been reported to impair axonal transport in dopaminergic neurons [57] and to deplete ATP content and antioxidant reserve [58], which could affect to a greater extent D2-cKO SNc DA neurons since they have a larger axonal compartment to maintain. Additionally, increased phosphorylation of α-synuclein to its pSyn-129 toxic form has been reported in the 6-OHDA model [59], which could play a role in the observed toxicity. However, it is unlikely that this effect on α-synuclein is the main mechanism leading to cell death in the present study because we failed to detect any change in vulnerability when we overexpressed α-synuclein, even if we observed the presence of pSyn-129 in surviving cell bodies.

This lack of an increased vulnerability to α-synuclein overexpression in the present model is presently unresolved, but it might be explained by the fact that pathology is initially induced in the cell bodies in this model, as opposed to its initiation in the terminals in the 6-OHDA model and that the time course of neurodegeneration is much longer in the overexpression model (months vs days for 6-OHDA). Additionally, the α-synuclein model is thought to trigger degeneration by causing pathological protein aggregation and impaired proteasome/lysosome function [60–62], unlike the 6-OHDA model, which directly impairs mitochondrial function by inhibiting mitochondrial complex I and IV and by inducing oxidative stress [63,64].

However, it has been suggested that α-synuclein overexpression can also influence mitochondrial function, but through different mechanisms. It has been proposed that once oligomerized, α-synuclein influences mitochondrial fusion/fission, transport, clearance and protein import mechanisms [65,66], as well as complex I and ATP-synthase function [67] and therefore increases oxidative stress [68]. Since α-synuclein oligomerization seems to be a necessary step for all these alterations, overexpression of WT α-synuclein should take much more time than 6-OHDA injections to elevate oxidative stress to critical levels. It should therefore also leave much more time for neurons to attempt to compensate for these changes compared to the 6-OHDA model where ATP and antioxidant depletion and oxidative stress are rapidly induced. In combination with the much more modest loss of striatal TH immunoreactive processes in the α-synuclein overexpression model, this could in part explain why behavioral alterations were almost absent in this model.

Additionally, it is also possible that the potentially enhanced level of oxidative stress in the nigro-striatal system of D2-cKO mice was not sufficiently elevated to promote enhanced vulnerability in response to all triggers of PD pathology. In line with this possibility, a global assessment of superoxide anion production and NADPH oxidase activity in the striatum and mesencephalon of the D2 cKO mice failed to reveal an increased stress level (S3 Fig). Further experiments would be needed to examine selective markers of oxidative stress in the axonal and somatodendritic compartment of DA neurons, without the confounding presence of signal originating in striatal neurons and glial cells.

Interestingly, even in the absence of exogenous triggers such as 6-OHDA or α-synuclein overexpression, features of PD pathophysiology such as loss of processes and presence of α-synuclein aggregates in the dorsal striatum have been reported in aged constitutive D2-KO mice [69]. In the present work, we did not produce nor examine aged D2-cKO mice, but it is possible that similar pathology would be observed.

In conclusion, this work demonstrates for the first time that SNc DA neurons in the intact brain possess a larger axonal arbour size compared to VTA DA neurons. This work also provides strong additional supportive evidence for the hypothesis that a very large axonal arbour places DA neurons at increased risk in PD.

## Methods

### Ethics statement

All procedures involving animals were conducted in strict accordance with the Guide to care and use of experimental animals (2nd Ed.) of the Canadian Council on Animal Care. The experimental protocols were approved by the animal ethics committee (CDEA) of the Université de Montréal.

### Animals

Housing was at a constant temperature (21°C) and humidity (60%), under a fixed 12h light/dark cycle and free access to food and water. Initial comparisons of the axonal arborization size of SNc and VTA DA neurons was performed using DAT-Cre knock-in mice [70]. The rest of the experiments were performed using DAT^IRES^cre mice obtained from Jackson Labs [71] and crossed with Drd2^loxP^ mice [48]. Mouse background was mixed 129SV/C57BL6 and both males and females were used.

### Genotyping

All animals were genotyped using a KAPA2G Fast HotStart DNA Polymerase kit from Kapa Biosystem. Primer used were:

DAT-Cre

        DAT-CRE up                        ACCAGCCAGCTATCAACTCG
        DAT-CRE lw                        TTACATTGGTCCAGCCACC

DAT^IRES^cre

        oIMR6625 Common                  TGG CTG TTG GTG TAA AGT GG
        oIMR6626 WTReverse              GGA CAG GGA CAT GGT TGA CT
          oIMR8292 Mutant Reverse     CCA AAA GAC GGC AAT ATG GT

Drd2^loxP^

             D2LOX-A#2                        GCT TCA CAG TGT GCT GCC TA
                D2LOX-B                           CCA TTG CTG CCT CTA CCA AG

### Axonal arborization labelling, α-synuclein and 6-OHDA lesions

Two-month-old DAT-Cre or DAT^IRES^cre positive mice were anesthetized with isoflurane (Aerrane; Baxter, Deerfield, IL, USA) and fixed on a stereotaxic frame (Stoelting,Wood Dale, IL, USA). Fur on top of the head was trimmed, and the surgical area was disinfected with iodine alcohol. Throughout the entire procedure, eye gel (Lubrital, CDMV, Canada) was applied to the eyes, and a heat pad was placed under the animal and kept warm. Next, bupivacaine (5 mg/ml and 2 mg/kg, Marcaine; Hospira, Lake Forest, IL, USA) was subcutaneously injected at the surgical site, an incision of about 1 cm made with a scalpel blade, and the cranium was exposed. Using a dental burr, one hole of 1 mm diameter was drilled above the site of injection [AP (anterior–posterior; ML (medial–lateral); DV (dorsal-ventral), from bregma]. The following injection coordinates were used:

- SNc for axonal arborization labelling [AP -3.0 mm; ML 1.5 mm; DV -4.0 mm]
- VTA for axonal arborization labelling [AP -2.7 mm; ML 0.0 mm; DV -4.5 mm]
- Mesencephalon for α-synuclein virus injection [AP -3.0 mm; ML 1.0 mm; DV -4.3 mm]
- Dorsal striatum for 6-OHDA injection [AP 0.5 mm; ML 2.0 mm; DV -3.0 mm].

Note that the coordinates for SNc and VTA injections were purposely 0.3 mm anterior to the center of the targetted region. These coordinates were adjusted to prevent infection of RRF, rostral linear nucleus (RLI) or caudal linear nucleus (CLI) DA neurons. Next, borosilicate pipettes were pulled using a Sutter Instrument, P-2000 puller, coupled to a 10 μL Hamilton syringe (Hamilton, 701RN) using a RN adaptor (Hamilton, 55750–01) and the whole setup was filled with mineral oil. Using a Quintessential Stereotaxic Injector (Stoelting), solutions to be injected were pulled up in the glass pipet. For the axonal arborization size quantification, 0.1 μL (SNc) or 0.05 μL (VTA) of sterile NaCl containing 1.15x10^12^ viral genome particles/mL of AAV2-EF1a-DIO-eYFP (UNC Vector Core, Chapel Hill, NC, USA) was injected. For α-synuclein over-expression, 0.8 μL of AAV2-CBA-alpha-Syn (3.8x10^12^ viral genome particles/mL, MJF Foundation, USA) or AAV2-CBA-eGFP (2.0x10^12^ viral genome particles/mL MJF Foundation, USA) was injected. For 6-OHDA lesions, 0.5 μL of 6-OHDA (3 mg/mL) in 0.2% ascorbic acid solution was injected. Forty minutes prior to 6-OHDA injections, the norepinephrine transporter blocker desipramine (25mg/Kg) was injected intraperitoneally to the animals to prevent lesions of the noradrenergic fibers. After the unilateral injection, the pipette was left in place for 10 min to allow diffusion and then slowly withdrawn. Finally, the scalp skin was sutured and a subcutaneous injection of the anti-inflammatory drug carprofen (Rimadyl, 50 mg/mL) was given. Animals recovered in their home cage and were closely monitored for 24h. A second dose of carprofen (5 mg/kg) was given if deemed necessary. The brains were collected 1 month after the 6-OHDA injection (P90), 2 months after viral injection for axonal arborization labeling (P120) or 3 months after viral injection for α-synuclein overexpression studies (P150).

### Tissue preparation for immunohistochemistry

Mice were anesthetized using pentobarbital NaCl saline solution (7 mg/mL) injected intraperitoneally and then were perfused with 50mL of PBS followed by 100 mL of paraformaldehyde (PFA) 4% using an intracardiac needle at a rate of 25 mL/min. The brains were extracted, placed 48h in PFA followed by 48h in a 30% sucrose solution and frozen in isopentane at -30°C for 1 minute. 40 microns thick coronal sections were then produced using a cryostat (Leica CM1800) and placed in antifreeze solution at -20 ^o^C until used.

### Immunohistochemistry

One out of every 6th slice was used for immunofluorescence. After a PBS wash, the tissue was permeabilized, nonspecific binding sites were blocked and slices were incubated overnight with a rabbit anti-TH antibody (1:1000, AB152, Millipore Sigma, USA), a rat anti-DAT antibody (1:1000, MAB369; MilliporeSigma, USA), a chicken anti-GFP (1:2000, GFP-1020; Aves Labs, USA), a mouse anti-p-S129-α-synuclein (1:2000, 328100, Invitrogen, USA), a chicken anti-α-synuclein (1:2000, AB190376, Cedarlane, USA) and/or rabbit anti-VMAT2 (1:2000, kindly provided by Dr. G.W. Miller [72]) Primary antibodies were subsequently detected with a rabbit or chicken Alexa Fluor-488–conjugated secondary antibody, a rabbit Alexa Fluor-546–conjugated secondary antibody, and/or a rat Alexa Fluor-647–conjugated secondary antibody (1:400; Thermo Fisher Scientific).

One out of every 6th slice was used for DAB immunostaining. After a PBS wash, the tissue was incubated for 10 min with 0.9% H_2_O_2_ solution, then washed with PBS again and incubated for 48h with a rabbit anti-TH antibody (1:1000, AB152, Millipore Sigma, USA) at 4°C, 12h with goat anti-rabbit biotin-SP-AffiniPure secondary antibody (111-065-003, Jackson ImmunoResearch Laboratories, USA) at 4°C and 3h with horseradish peroxidase streptavidin (016-030-084, Cedarlane, USA). The DAB reaction was carried out for 45s, then stopped by incubation with 0.1M acetate buffer and slices were mounted on Superfrost/Plus microscope slides. They were left to dry for 96h after which they were stained with cresyl violet and went through subsequent incubations with increasing concentrations of alcohol. After short isopropanol and xylene baths, slides were sealed with Permount mounting medium (SP15-100, Fisher, USA) using glass coverslips.

### Confocal imaging

All of the imaging quantification analyses were performed on images captured using confocal microscopy. Images were acquired using an Olympus Fluoview FV1000 microscope (Olympus). Images acquired using 488 and 546 nm laser excitation were scanned sequentially to reduce nonspecific bleed-through signal. For each slice, up to 4 images were acquired in the dorsal striatum and up to 2 in the ventral striatum. All image quantifications were performed using ImageJ (National Institutes of Health) software. We first applied a background correction and then measured the area and intensity of the signal.

For quantification of TH, DAT and VMAT2 positive terminals in the ventral or dorsal striatum, images were acquired using a 60x oil-immersion objective and averaged from slices at bregma 1.18, 0.14 and -0.94 mm. For axonal arborization size quantification with eYFP viral expression, images were acquired on one out of every 6th slice from bregma -2.2 to 1.94 mm using a 20x water immersion objective since the fibers were easily distinguishable at lower magnification. The proportion of the area covered by eYFP fibers was extrapolated to the size of the striatum for each slice based on The Mouse Brain in Stereotaxic Coordinates 3rd Edition by George Paxinos [73] normalized by the number of infected neurons counted manually (300–1000 neurons) and plotted in relation to the bregma coordinates. Stereological counting was not used for this quantification since the number of neurons was too low to get a reliable count using random sampling. The volume of eYFP positive axonal arborization was then approximated using the area under the curve.

The number of striosomes and their size was also quantified using the integrated particles analyzer in Image J. Colocalization measurements were performed using the Jacop plugin for ImageJ on 60x confocal images [74]. Mander’s M1 and M2 coefficients were obtained after manual thresholding of the images to remove background. A mask of the YFP signal was applied to the other signals for measurement of their colocalization inside YFP fibers.

### Stereological counting

TH-immunoreactive neurons were counted in one out of every sixth section using a 100x oil-immersion objective on a Leica microscope equipped with a motorized stage. A 60 x 60 μm^2^ counting frame was used in the Stereo Investigator (MBF Bioscience) sampling software with a 12 μm optical dissector (2 μm guard zones) and counting site intervals of 150 μm after a random start (100 μm intervals for unilateral lesion). Mesencephalic DA nuclei, including the VTA, SNc and RRF were examined. Stereological estimates of the total number of TH-immunoreactive neurons within each nucleus were obtained. The number of TH-negative neurons was also estimated similarly in each region based on cresyl violet staining.

### Fast scan cyclic voltammetry

Acute brain slices from 3-month-old mice were obtained using a protective slicing method [75]. Matched pairs of CTL and D2-cKO mice were used on each experimental day. After intracardiac perfusion, brains were quickly dissected, submersed in ice-cold NMDG cutting solution and coronal striatal brain slices of 300 μm (from bregma AP 1.34 to 0.98 mm) were prepared with a Leica VT1000S vibrating microtome in ice-cold (0 to 4°C) NMDG protective cutting solution. Slices recovered for 12 min in 32° NMDG solution and were then transferred to oxygenated HEPES-buffered resting solution at RT for at least 1h. For recordings, slices were put in a custom-made recording chamber superfused with artificial cerebral spinal fluid (aCSF) at 1 mL/min and maintained at 32°C. All solutions were adjusted at pH 7.35–7.4, 300 mOsm/kg and saturated with 95% O_2_-5% CO_2_ at least 30 min prior to each experiment.

Electrically induced DA release was measured by fast-scan cyclic voltammetry (FSCV) using a 7 μm diameter carbon-fiber electrode placed into the dorsal or ventral striatum ∼100 μm below the surface and a bipolar electrode (Plastics One, Roanoke, VA, USA) placed ∼200 μm away. Carbon-fiber electrodes were fabricated as previously described [76]. Electrodes were polished and filled with 4M potassium acetate and 150 mM potassium chloride. Carbon fibers were then cut using a scalpel blade to obtain maximal basal currents of 100 to 180 nA. Electrodes were finally selected for their sensitivity to DA using *in vitro* calibration with 1μM DA in aCSF before each experiment. Before and after use, electrodes were cleaned with isopropyl alcohol. The potential of the carbon fiber electrode was scanned at a rate of 300 V/s according to a 10 ms triangular voltage wave (−400 to 1000 mV vs Ag/AgCl) with a 100 ms sampling interval, using a CV203BU headstage preamplifier (Axon instrument, Union City, CA)) and an Axopatch 200B amplifier (Axon Instruments). Data were acquired using a Digidata 1440A analog to digital converter board (Axon Instruments) connected to a computer using Clampex (Axon Instruments). Slices were left to stabilize for 20 min before any electrochemical recordings.

After positioning of the bipolar stimulation and carbon fiber electrodes in the striatum, single pulses (400 μA, 1ms) were applied to the nucleus accumbens core (referred to as ventral striatum) and then to the dorso-lateral part of the dorsal striatum to trigger DA release. Stimulations were applied every 2 min. After recording in the dorsal striatum, the media was changed to ACSF containing 5 μM of nomifensine (Sigma) and single stimuli were applied to the dorsal striatum. Electrode calibration was performed before and after the recording of each slices and the average value for the current at the peak oxidation potential was used to normalize the recorded *ex vivo* current signals to DA concentrations.

DA release was analyzed as the peak height of DA concentrations and DA reuptake was determined from the clearance rate of DA which was assumed to follow Michaelis-Menten kinetics. A nonlinear least square optimization was applied to fit a three-parameter exponential function with baseline shift to the reuptake phase of the DA response. Uptake parameters (tau and Vmax) were calculated based on the exponential fitting. To determine whether DAT-mediated DA uptake was compromised in D2-cKO mice, the initial portion of the falling phase of single pulse evoked [DA]o curves was used to calculate the Vmax (maximal rate of DA uptake) after setting the Km parameter to 0.2 μM, based on the affinity of DA for the DAT, measured in mouse synaptosome preparations [77] and with the assumption that the Km is not altered in the KO mouse line.

### DAT surface biotinylation assay

Surface biotinylation experiments were carried using a protocol modified from Rickhag et al. 2013 [78]. Brains from 3-month-old conditional D2-cKO mice and CTL littermates were rapidly dissected and submerged in pre-oxygenated (95% O_2_ and 5% CO_2_) ice-cold sucrose buffered artificial cerebrospinal fluid. Coronal striatal sections (300 μm) were obtained using a vibrating blade microtome (Leica VT1000). The slices were allowed to recover in oxygenated aCSF (without sucrose) for 1h at room temperature. After surface biotinylation, slices were rinsed twice and excess biotin was quenched by two washes in glycine in oxygenated aCSF (4°C). The biotinylated slices from individual mice were pooled and homogenized in lysis buffer containing protease and phosphatases inhibitor. The homogenates were quickly incubated, gently mixed and centrifuged to remove debris (4°C).

Protein concentrations were measured and adjusted to 1ug/ml, and 100 μl of total lysates were stored to allow determination of the total protein input. Biotinylated proteins were isolated by loading equal amounts of protein onto 175ul avidin beads (Thermo Scientific) followed by overnight incubation at 4°C. Beads were washed in lysis buffer before elution of biotinylated proteins. Avidin beads were removed by filtration, and surface and total DAT levels were evaluated by western blot analysis. Protein samples were separated by SDS-PAGE and transferred to membranes. The membranes were blocked and then incubated subsequently with antibodies against DAT (Millipore MAB369, 1:1000) and with horseradish peroxidase (HRP)-conjugated anti-rat antibodies. Surface DAT protein bands were visualized by chemiluminescence. Blots of surface protein samples were reprobed for Na^+^/K^+^-ATPase (Abcam 1:500) to account for variation in biotinylated input while actin (HRP-conjugated actin (1:10000, A3854, mouse monoclonal, Sigma) was used as loading control for the total lysates. Band intensities were quantified using ImageJ gel analysis software.

### Determination of superoxide anion production and NADPH oxidase activity

Basal superoxide anion production and NADPH oxidase activity in brain tissues were measured using the lucigenin‐enhanced chemiluminescence method with a low concentration (5 μmol/L) of lucigenin, as described previously [79]. The tissues from control and D2-cKO mice were washed in oxygenated Krebs HEPES buffer and placed in scintillation vials containing lucigenin solution, and the emitted luminescence was measured with a liquid scintillation counter (Wallac 1409; Perkin Elmer Life Science) for 10 minutes. The average luminescence value was estimated, the background value was subtracted, and the result was divided by the total weight of tissue in each sample. The NADPH oxidase activity in the samples was assessed by adding 10 to 4 mol/L NADH (Sigma‐Aldrich) in the vials before counting. Basal superoxide–induced luminescence was then subtracted from the luminescence value induced by NADH.

### Behavior testing

All mice were habituated to the user by handling them once a day during 3 consecutive days before experiments. Mice were moved to the experimental room 1h before the test.

Mice first went through a stepping test recorded with a digital camera (DMK 22BUC03, ImagingSource) and IC Capture 2.4 software. Mice were gently lifted by the base of the tail at one end of a 1-meter corridor leaving only forepaws touching the surface and were pulled backward for 4s over a distance of 1-meter. Recordings were then watched in slow motion and the number of steps of each forepaw was counted. After 1h of rest, animals were placed in a 4L beaker with the digital camera recording their movements from underneath to assess rotation. After 20 min, amphetamine 5 mg/kg was intraperitoneally injected and mice were placed back in the beaker for 40 min. Recordings were then watched to count the ipsilateral and contralateral rotations made by the mice during the first (basal) and the last (amphetamine) 20 min.

### Statistics

All experiments were performed blind to the experimental groups, from surgeries to image analysis. Parametric statistical tests were used because samples contained data with normal distributions. Data were presented as mean ± SEM. The level of statistical significance was established at *p* < 0.05 in one or two-way ANOVAs or two-tailed t-tests with Welch’s correction when needed. A ROUT outlier analysis was performed when required (Q = 1%). Statistical analyses were performed with the Prism 7 software (GraphPad Software, *p* < 0.05 = *, *p* < 0.01 = **, *p* < 0.001 = ***, *p* < 0.0001 = ****). The Tukey post-hoc test was used when all the means were compared to each other and the Sidak post-hoc test was used when only subsets of means were compared.

## Supporting information

S1 Fig Example of AAV infection in 6 adult DAT^IRES-Cre^ mice to conditionally express eYFP in SNc (general area represented by red dash line). Infected DA neurons were visualized by immunofluorescent labelling for eYFP. Scale bar = 300 μm.
(EPS)

S2 Fig Behavioral changes following α-synuclein overexpression. Paw preference (A) and total number of steps (B) were measured during a stepping test. Rotational behaviour was assessed at basal state (C) and after amphetamine administration (D). Total number of rotations were also measured (E). N = 4–11 brains/group, mean ± SEM, ** p>0.01, **** p>0.0001.
(EPS)

S3 Fig Global assessment of oxidative stress in the striatum and mesencephalon of the D2-cKO mice. Superoxide production was measured in tissue extracts of the striatum (A) and mesencephalon (B) of a separate cohort of control and D2-cKO mice, followed by measurements of NADPH oxidative activity in extracts from the same regions (C,D). N = 6–9 brains/group, mean ± SEM.
(EPS)
